# Biogeographical patterns of the soil fungal:bacterial ratio across France

**DOI:** 10.1128/msphere.00365-23

**Published:** 2023-09-27

**Authors:** Christophe Djemiel, Samuel Dequiedt, Arthur Bailly, Julie Tripied, Mélanie Lelièvre, Walid Horrigue, Claudy Jolivet, Antonio Bispo, Nicolas Saby, Matthieu Valé, Pierre-Alain Maron, Lionel Ranjard, Sébastien Terrat

**Affiliations:** 1 Agroécologie, INRAE, Institut Agro, Université Bourgogne, Franche-Comté, Dijon, France; 2 INRAE, Info&Sols, Orléans, France; 3 AUREA AgroSciences, Ardon, France; University of Wisconsin-Madison, Madison, Wisconsin, USA

**Keywords:** soil microbiology, molecular biology, microbial ecology, bioindicators

## Abstract

Soils are one of the major reservoirs of biological diversity on our planet because they host a huge richness of microorganisms. The fungal:bacterial (F:B) ratio targets two major functional groups of organisms in soils and can improve our understanding of their importance and efficiency for soil functioning. To better decipher the variability of this ratio and rank the environmental parameters involved, we used the French Soil Quality Monitoring Network (RMQS)—one of the most extensive and a priori-free soil sampling surveys, based on a systematic 16 km × 16 km grid and including more than 2,100 samples. F:B ratios, measured by quantitative PCR targeting the 18S and 16S rDNA genes, turned out to be heterogenously distributed and spatially structured in geographical patterns across France. These distribution patterns differed from bacterial or fungal densities taken separately, supporting the hypothesis that the F:B ratio is not the mere addition of each density but rather results from the complex interactions of the two functional groups. The F:B ratios were mainly influenced by soil characteristics and land management. Among soil characteristics, the pH and, to a lesser extent, the organic carbon content and the carbon:nitrogen (C:N) ratio were the main drivers. These results improved our understanding of soil microbial communities, and from an operational point of view, they suggested that the F:B ratio should be a useful new bioindicator of soil status. The resulting dataset can be considered as a first step toward building up a robust repository essential to any bioindicator and aimed at guiding and helping decision making.

**IMPORTANCE:**

In the face of human disturbances, microbial activity can be impacted and, e.g., can result in the release of large amounts of soil carbon into the atmosphere, with global impacts on temperature. Therefore, the development and the regular use of soil bioindicators are essential to (i) improve our knowledge of soil microbial communities and (ii) guide and help decision makers define suitable soil management strategies. Bacterial and fungal communities are key players in soil organic matter turnover, but with distinct physiological and ecological characteristics. The fungal:bacterial ratio targets these two major functional groups by investigating their presence and their equilibrium. The aim of our study is to characterize this ratio at a territorial scale and rank the environmental parameters involved so as to further develop a robust repository essential to the interpretation of any bioindicator of soil quality.

## INTRODUCTION

Soil microbial communities play essential roles in nutrient cycling and ecosystem productivity as principal decomposers of organic matter (1, 2). Bacterial and fungal communities largely control the soil organic matter turnover, but with distinct physiological abilities regarding soil functions (2, 3). In bacterium-dominated soils, organic matter decomposition and nutrient mineralization are fast, while the conversion rates of nutrients and energy are relatively slow in fungus-dominated soils (3). Given the functional complementarity of these two microbial groups in soil functioning, it makes sense that metrics—considered as bioindicators—should be available to investigate their presence and/or equilibrium. The fungal:bacterial (F:B) ratio is one of them (4).

The F:B ratio has been widely used for a decade in the context of land management and its effects on soil carbon sequestration (2) or to investigate the importance of microbial communities as a predictor of soil resistance to climate change (5). It has high ecological significance in soil functioning (e.g., litter decomposition) and plant productivity (6) and may reflect the self-regulation ability of soil ecosystems (3). Numerous approaches, like phospholipid fatty acids (PLFAs), substrate-induced respiration, or quantitative PCR (qPCR) have been used to determine F:B ratios following the gradual development of methodologies for microbial studies, each with their own drawbacks and advantages (2). These quantification techniques have been extensively used, but also compared (7). Despite differences, they have altogether showed good repeatability. However, there is a clear need to standardize methods like the DNA extraction procedure for qPCR (8) or specific biochemical steps for PLFA analyses (7) to obtain comparable results across studies or laboratories and evaluate the biological status of soils (9). A first recent meta-analysis going into this direction focused on 1,323 measurements of the soil F:B ratio using PLFA analyses among 11 major biomes to produce the first global maps of F:B biomass ratios (10). These authors showed clear spatial patterns on a global scale. The F:B ratio varied from 1.8 in savanna to 8.6 in tundra on average, mainly driven by climatic variables and edaphic properties (including the clay content, soil moisture, or the soil organic carbon content). Such global studies are quite informative to territorial-scale conservation managers but did not result in the development of a new bioindicator of the soil ecological quality (11). The soil ecological quality—defined as the capacity of a soil to host a great quantity and diversity of interacting living organisms implied in its functioning—has been intensively studied as a consequence of the current need for an agroecological transition of soils and of the development of modern research tools (11, 12). There is now a clear need to have access to robust molecular tools to efficiently reflect the complex interactions of the bacterial and fungal soil microbial communities (9), particularly in agricultural soils such as croplands or managed grasslands.

To better decipher the variability of the F:B ratio at the territorial scale and rank the environmental parameters involved in it, we used the French Soil Quality Monitoring Network (RMQS) (13). This survey captures various land uses—mainly agricultural soils (like grasslands, croplands, and vineyards & orchards)—very distinct climates, a wide range of geomorphology types and soil characteristics concentrated on a small territory (544,103 km^2^) across France. Furthermore, this survey had been used to characterize and develop other soil microbial bioindicators such as soil molecular biomass (14) or soil bacterial richness (15). We chose to expand our analyses to determine the F:B ratios of RMQS soil samples by qPCR targeting the 18S and 16S rDNA genes to improve our understanding of soil microbial communities, as a first step toward defining a new molecular bioindicator of soil ecological quality.

## RESULTS

**FIG 1:**
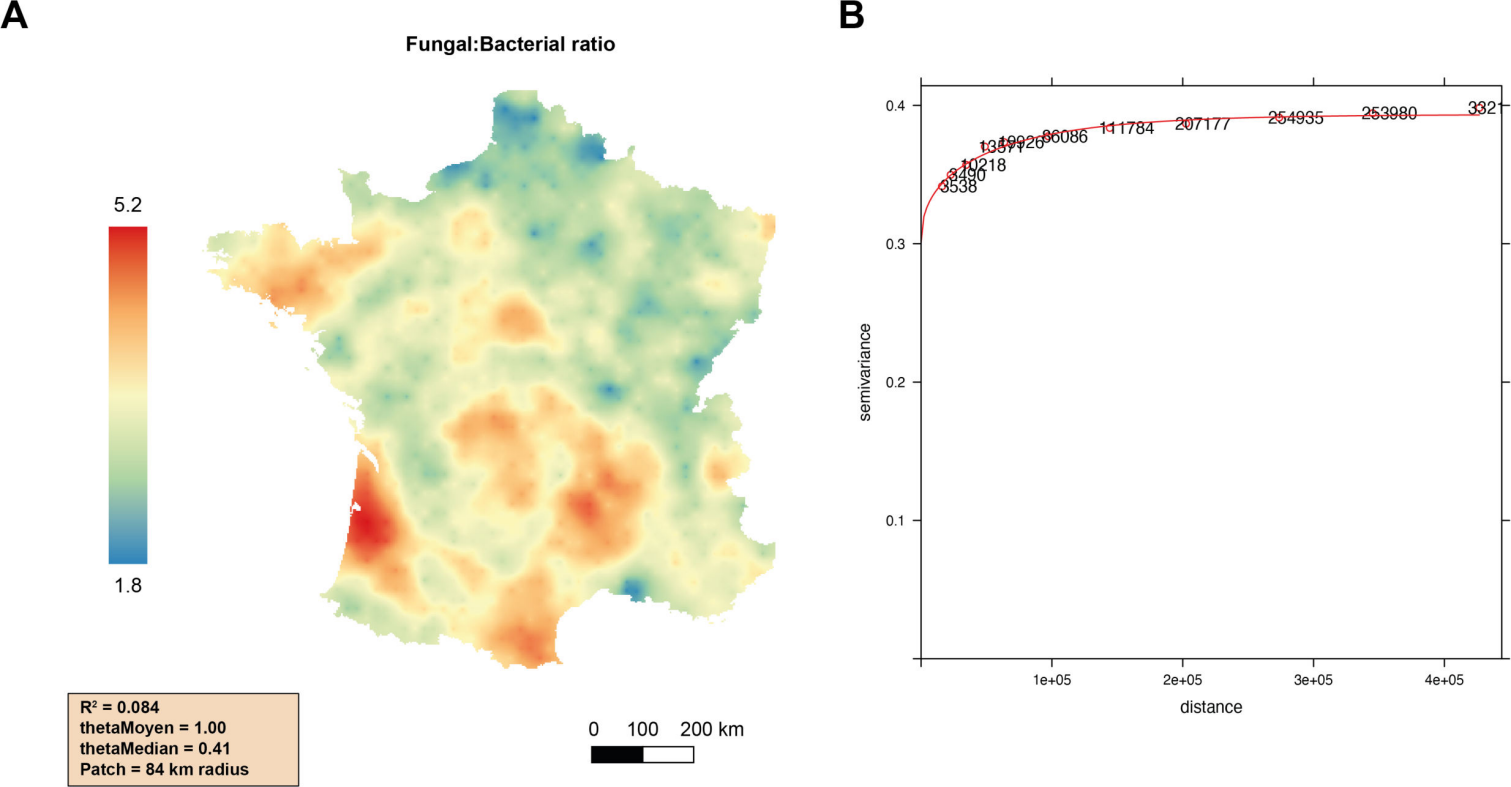
Mapping (**A**) and theoretical variograms (**B**) of soil F:B ratios at the scale of mainland France. The colors indicate the extrapolated values expressed as F:B ratio (18S rDNA copy number per gram of soil/16S rDNA copy number per gram of soil × 100) per soil sample (**A**). The quality parameters of the model are also detailed. For the variograms (**B**), points represent the experimental variogram values, and continuous lines represent the Matern models fitted by the maximum likelihood method.

F:B ratios across France ranged from 0.24 to 12.15 (mean 3.29, median 2.72), and a majority of values ranged between 1 and 5 (Text S1 in the supplemental material). Geostatistical analyses revealed a heterogenous distribution of F:B ratios, spatially structured in geographical patterns with an autocorrelation distance of around 84-km radius and *R* ^2^ = 0.08 (Fig. 1). Four regions displayed higher F:B ratios: southwestern France (Landes), the western Mediterranean coast, the Massif Central, and Brittany. On the opposite, two regions—northern and northeastern France—exhibited lower F:B ratios. Other French regions had medium, rather homogenous F:B ratios, yet with hotspots and coldspots, as on the central and eastern Mediterranean coast (Fig. 1). Interestingly, the F:B ratio map clearly differed from the maps of bacterial and fungal densities (Fig. S1 and S2).

**FIG 2:**
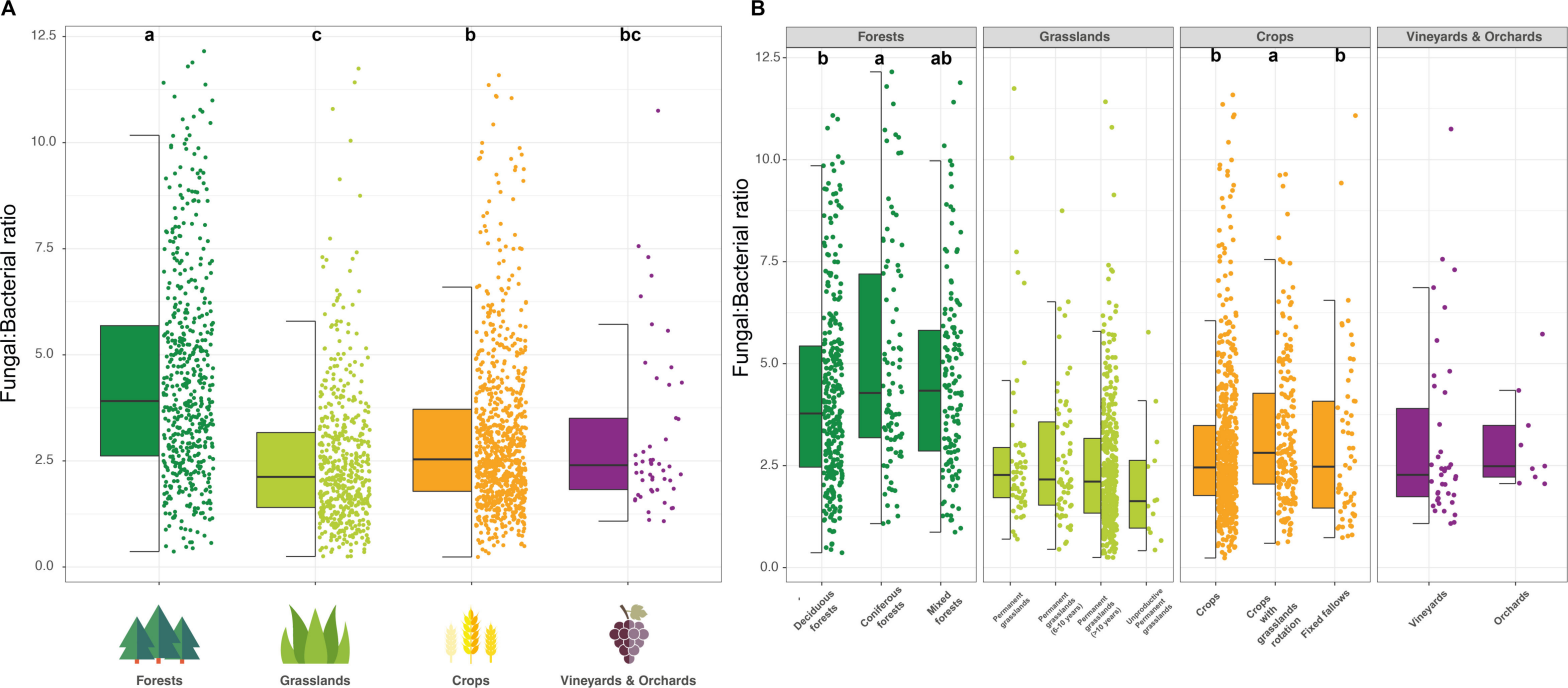
Distribution of F:B ratio values for coarse (**A**) and more precise (**B**) land uses. The colors indicate the global land uses (forests, grasslands, crops, and vineyards & orchards). Significant differences are indicated after Kruskal-Wallis tests with Bonferroni correction (*P* value < 0.05).

Comparing this ratio based on the different land uses, significant dissimilarities were observed (Kruskal-Wallis tests with Bonferroni correction, *P* value < 0.05) (Fig. 2A). The lowest F:B ratios were found in grassland soils (median 2.12) and the highest ones in forest soils (median 3.91). Other land uses (crop systems and vineyards & orchards) exhibited significantly lower ratios than those of forests (medians = 2.54 and 2.40, respectively). Most F:B ratios ranged between 1 and 5, with differences between land uses: forests harbored higher ratios (9.69% of soils with ratios >5) than more anthropized soils such as grasslands or crops did (5.5% of soils with ratios <1) (Text S1B). Our results also revealed significant differences in bacterial density (Kruskal-Wallis tests with Bonferroni correction, *P* value < 0.05) (Fig. S3A). The highest values were observed from grassland soils, the lowest ones from vineyard & orchard soils, and average ones from forests and crop systems. Fungal density was distributed differently between the major soil managements (Fig. S4A). The highest values came from natural or semi-natural environments (forests and grasslands), and the lowest values came from agricultural soils (crops and vineyards & orchards).

A comparison of F:B ratios within each land use showed that a majority of ratios ranged from 1 to 5, except for less managed soils like forests, where they were higher (see Text S2). Significant differences were highlighted between deciduous forests (median 3.78) and coniferous forests (median 4.28), and between crops with grassland rotation (median 2.81) and crops without rotation or fixed fallows (medians 2.46 and 2.47, respectively) (Kruskal-Wallis tests with Bonferroni correction, *P* value < 0.05) (Fig. 2B; Text S2B). These differences between crop systems were also visible for fungal density, but no significant difference in bacterial density emerged at this level of analysis (Fig. S3B and S4B). Conversely, the F:B ratios of grasslands and vineyards & orchards were statistically similar (Fig. 2B).

**FIG 3:**
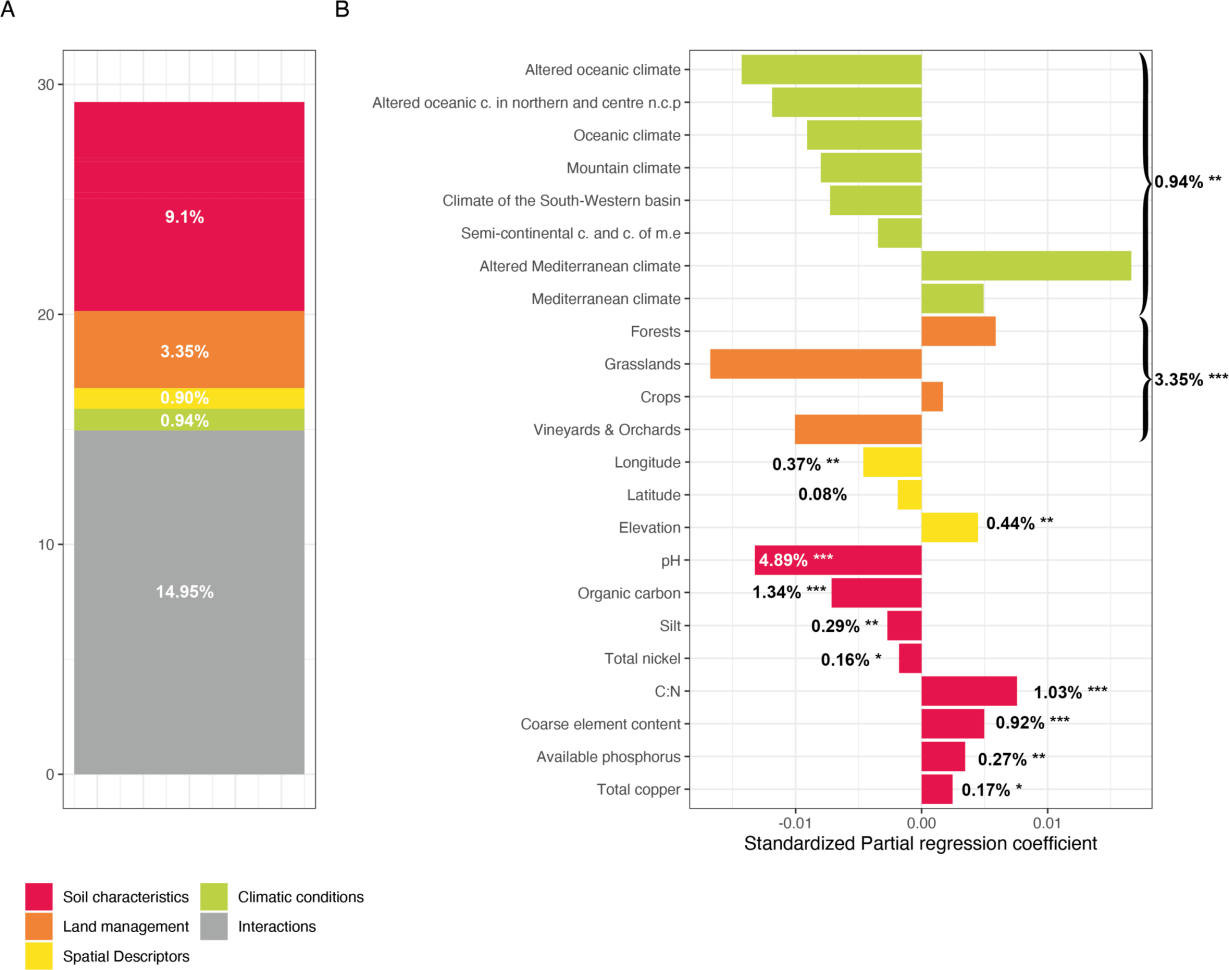
Variance partitioning analysis to determine how local factors and factors related to global environmental filters explained the variance of F:B ratios. (**A**) Explained variance of F:B ratios across France. The amount of explained variance corresponds to the adjusted *R* ^2^ values of the contextual groups using partial redundancy analysis. (**B**) Model parameters for the distribution of F:B ratio values. Each parameter is presented with its estimated model coefficients and its marginal effect was assessed by a permutation test. *P < 0.1, **P < 0.01, ***P < 0.001. Missing values indicate that the variable was not retained in the model. Sand was removed prior to model evaluation because it was represented by the opposite of the sum of the silt and clay contents.

A variance partitioning approach of F:B ratios revealed a total amount of explained variance of 29.2% with a significant influence of soil characteristics (9.1%), land management (3.3%), and climatic conditions and spatial descriptors to a lesser extent (0.94% and 0.9%, respectively) (Fig. 3A; Table S1). Interactions between land uses, climatic conditions, and soil properties also represented a large proportion of the explained variance (14.95%). Regarding soil characteristics, the F:B ratio was negatively impacted by the pH (4.89%, *P* value = 0.001) and the organic carbon content (1.34%, *P* value = 0.001), but positively impacted by the carbon:nitrogen (C:N) ratio (1.03%, *P* value = 0.001) and the coarse element content (0.92%, *P* value = 0.001) (Fig. 3B). Other soil parameters [silt (*P* value = 0.01), total nickel (*P* value = 0.039), available phosphorus (*P* value = 0.006), and total copper (*P* value = 0.035)] had a significant but marginal effect. F:B ratios were also positively impacted by forests and crops, and negatively impacted by grasslands and vineyards & orchards, depending on the signs of the standardized estimated coefficients of land management categories (Fig. 3B). Finally, only Mediterranean climates had a positive effect on F:B ratios among all defined climatic conditions.

Bacterial densities harbored higher explained variance (41.4%) than the F:B ratios did, with a significant influence of the soil characteristics (16.7%) and interactions (22.9%) (Table S1). The same trends were measured for fungal densities (Table S1). Among the soil characteristics, the organic carbon content influenced bacterial and fungal densities positively (11.13% and 7.45% [*P* value = 0.001], respectively) while the C:N ratio influenced them negatively (3.25% and 2.24% [*P* value = 0.001], respectively). The pH (*P* value = 0.001) was a less major driver of the sole bacterial densities. Land management and climatic conditions also explained a lesser part of the variance of bacterial and fungal densities, and only fungal densities were influenced by spatial descriptors (1.07%) (Table S1).

## DISCUSSION

The quantification of microorganisms in a complex environment is a crucial step for estimating the absolute abundance of each microbial group and their equilibrium. Few studies have compared PLFA measurements with qPCR methods in the estimation of absolute microbial quantities, but some comparisons showed good repeatability (7). Although PLFA profiles are widely used to measure microbial biomass and community composition in soils and other types of environmental samples, PLFA analysis is somewhat slow and expensive to carry out (7), particularly for a high diversity of soil physico-chemical characteristics. This is why we based our analytical strategy on qPCR after careful consideration of existing biases. The molecular strategy presents high levels of robustness despite well-known biases inherent in qPCR like the DNA extraction (8, 16), the primer choice (17), the amplification process (18), and copy number variation across bacterial and fungal species. For example, extracting DNA from microbial communities from a complex matrix like a soil is a challenging task (18). This has led to the development of numerous custom DNA extraction protocols as well as commercial kits, each with its own advantages and potential biases (8, 18). We used an improved and standardized soil DNA extraction procedure that gives a good snapshot of bacterial and fungal communities, previously evaluated by qPCR and by metabarcoding (8, 16). To keep our methodology efficient and robust, we also used recognized primers with high coverage rates to detect most of both microbial communities (17, 19). However, problems can still impact the results, like copy gene number variation across bacterial and fungal species (20). Many bacteria and fungi have more than one copy of the targeted gene, which leads to biased cell count estimates, particularly for fungi that are multinucleate cells with very variable numbers of nuclei per cell, depending on the species (17, 20). Even though tools and methods have been proposed to manage these discrepancies, the prediction of gene copy numbers for the large number of clades whose genomes are unsequenced will generally be inaccurate for close relatives (20). This is why the abundances of the two soil microbial communities estimated from either 16S or 18S rRNA gene copy numbers were not converted into numbers of bacterial/fungal cells per gram of soil.

France covers a relatively small area (0.3% of terrestrial surface on Earth) but exhibits a wide range of land managements and a high diversity of soils and climates (21). Therefore, it represents an excellent broad-scale playground to address the spatial distribution of soil F:B ratios, identify the ranges of variation, and rank the environmental filters involved.

The computed map of F:B ratios across France revealed a heterogenous, spatially structured distribution, with short biogeographical patches of 84-km radius. Compared to the recent global-scale distribution of F:B biomass ratios based on PLFA measurements (10), we highlighted regional differences more precisely, thanks to the intensive sampling survey of the French territory based on a systematic grid (16 km × 16 km). This observation stresses the need to assess more exhaustive sampling surveys at smaller scales than a global one to efficiently describe F:B ratio variation and its environmental drivers. Moreover, the distribution patterns of the F:B ratio maps differed from the previously described patterns of microbial molecular biomass and 16S richness maps (14, 15), with hotspots of microbial molecular biomass in the southern mountainous area (the Pyrenees), central and eastern France, and hotspots of bacterial richness in Brittany, the Mediterranean coast, and northern France. More importantly, the F:B ratio also exhibited different and specific biogeographical patterns compared to bacterial or fungal density maps taken separately. The organic carbon content and the C:N ratio were indeed the main drivers of the soil fungal and bacterial densities taken separately (highlighting the amount and quality of C substrates available for microbial development), but the relative contributions of bacteria and fungi differed, as evidenced by variance partitioning (Table S1). As a consequence, the F:B ratio reflected the complex interactions of these two different functional groups—another key aspect of the understanding of soil microbial communities (9). Taken together, these results evidence that the F:B ratio provides good complementarity information regarding bacterial and fungal communities, both implied in soil functioning. Therefore, the F:B ratio can be used as a soil ecological quality bioindicator when determined together with other bioindicators such as microbial biomass or diversity.

Among the soil characteristics, the pH, the organic carbon content to a lesser extent, and the C:N ratio were the main drivers of the F:B ratio in our analysis. The pH influenced the F:B ratio, but not fungal density, in line with previous results (22) of a long-term experiment in which variations of other factors than the pH were minimized. Fungal communities typically harbor a wider pH optimum than bacterial communities, without significant inhibition of their growth (23). The positive effect of the C:N ratio on the F:B ratio can be related to the ecology of each of its two functional members. The high F:B ratios found in high C:N soils can be explained by the fact that saprotrophic fungi have a more efficient enzymatic machinery than bacteria to decompose complex organic material and have lower nitrogen requirements than bacteria (24, 25).

Interestingly, other environmental parameters seemed to impact the F:B ratio to a lesser extent. For example, the coarse element content positively influenced the F:B ratio and fungal density but did not influence bacterial diversity. Compared to bacteria, fungal cells are generally much larger and can form “hyphae” and long “mycelia” in soils. Moreover, coarse-textured soils offer a more favorable habitat for fungal growth because plant litter fragments located in larger soil pores are more easily accessible (26, 27). This is particularly the case when the microenvironments are rich in bioavailable substrates formed by fresh unprotected litter (27). The filamentous growth of the “mycelium” enables many fungi to bridge air-filled pore spaces to adapt to heterogenous pore networks (28). Consequently, under the physical conditions of soils with a high coarse element content, fungi have a clear advantage over other microorganisms to grow rapidly. This is also in agreement with our results regarding the negative impact of the silt content—considered as fine particles—on the F:B ratio and fungal density.

Moreover, available phosphorus was also a driver of the F:B ratio and fungal density. This is consistent with recent results obtained with a PLFA approach on a continental-scale analysis of soils (29). Fungi play an important role in nutrient cycling in soil ecosystems by facilitating nutrient availability via mineralization or directly transporting nutrients through hyphal networks (30). However, the response to available phosphorus differed between fungal groups, not discriminated with fungal density measurements. For example, enhanced phosphorus availability for plants is often attributed to arbuscular mycorrhizas (31), but other fungi can be favored by phosphorus addition. This phosphorus addition, e.g., to various grassland soils, can promote the relative abundance of fungal pathogens and suppress mutualists without affecting saprotrophs (32).

A significant effect of land use on the F:B ratio was also demonstrated. Measured values were higher in forests, particularly in coniferous forests (environments less submitted to human disturbances like tillage and/or fertilization), in line with the previous assumption that soils with complex organic material (i.e., a high C:N ratio, with aromatic substrates) are more advantageous ecosystems for fungi than for bacteria (24, 25). F:B ratios were also higher in crops incorporating year-round vegetation with grassland rotation, potentially explained by an increasing plant diversity and consequently higher fungal diversity and density (33). Contrastingly, soils with lower F:B ratios, such as more intensively managed crop or vineyard & orchard soils (e.g., impacted by tillage and/or fertilization), exhibited lower fungal density, while bacteria were favored (3, 34). Additionally, agricultural practices favoring lower F:B ratios can be responsible for more carbon dioxide emissions through respiration, as carbon storage is thought to be more persistent in soils where decomposition is mainly mediated by fungi (35, 36).

Our results altogether emphasize that the F:B ratio can be linked to important features of the ecological significance of soil functioning such as soil carbon cycling (35) or nutrient availability (31).

### Conclusion

Fungi and bacteria—main decomposers of soil organic matter—display different metabolisms and life strategies. They are impacted by soil environmental parameters differently than the F:B ratio that describes the equilibrium and/or the dominance of these two major functional groups of microorganisms. Our results show high correlations of F:B ratios with specific environmental parameters like the pH or the C:N ratio, but also a clear influence of land use, suggesting that F:B ratios should be useful as a new bioindicator of the soil status. The data set obtained in the present study can be considered as a first step toward elaborating a robust repository essential to the interpretation of any bioindicator. F:B ratio measurements combined with other validated microbial bioindicators such as molecular microbial biomass or bacterial richness should be helpful to better evaluate soil ecological quality. Microbial data from well-documented spatial soil monitoring surveys at different large scales offer a great potential to (i) improve our knowledge of soil microbial communities and (ii) build robust indicators to guide and help decision makers in defining suitable soil management strategies. Given the importance of the soil microbiome in determining the one-health components (plant, animal, human, and ecosystem), governments should initiate and support systematic monitoring tools to investigate the trends, threats, and long-term developments of the soil microbiome.

## MATERIALS AND METHODS

### Soil sampling design

Soil samples were obtained from the RMQS, a soil monitoring network based on a systematic random sampling following a regular 16 × 16 km grid across the 544,000 km^2^ French mainland territory (13). The RMQS included 2,171 monitoring sites, each located at the center of a 16 × 16 km cell whose soil profile, site environment, climatic factors, localization, vegetation, and land management were described. All details concerning soil sampling and storage have been deposited (https://www.gissol.fr/publications/french-soil-quality-monitoring-network-manuel-rmqs2-edition-2018-english-version-5968), and details about physico-chemical analysis can be found in references 15 and 37 .

### Molecular characterization of microbial communities

#### Sample preparation

Microbial DNA was extracted from 1 g of each of the 2,171 composite soils using the GnS-GII procedure (16), and DNAs were purified using a Nucleospin soil kit (Macherey-Nagel) and quantified by fluorescence (QuantiFluor, Promega) using a Tecan Infinite f200 pro microplate reader, and then normalized to 1 ng/µL.

#### Determination of microbial density by quantitative PCR

Specific primers were used for the real-time PCR to quantify bacterial densities (341F: 5′-CCTACGGGAGGCAGCAG-3′ and 515R: 5′-ATTACCGCGGCTGCTGGCA-3′) and fungal densities (FR1: 5′-ANCCATTCAATCGGTANT −3′ and FF390: 5′-CGATAACGAACGAGACCT-3′) (8, 17). Quantitative real-time PCR was performed with a SYBR Green detection system in a total reaction volume of 15  µL, containing 1 µM of each primer (Eurogentec, Belgium) for bacterial densities and 1.25 µM of each primer (Eurogentec, Belgium) for fungal densities, 0.5 µg of T4 gene 32 protein (MPbio, Santa Ana, CA, USA), 1× SYBR Green Master Mix (Takyon qPCR kits, Eurogentec, France), 2 ng of DNA template, and DNase-free water to make up a final volume of 15 µL. Before water addition, more MgCl_2_ was added to the reaction mixture for fungal densities (0.7 µL of 50-mM solution).

Real-time PCR conditions for bacterial densities consisted in an initial denaturation step of 15 min at 95°C to activate the enzyme, then a second step of 35 cycles comprising a denaturation step of 15 s at 95°C, a primer annealing step of 30 s at 60°C, a final extension step of 30 s at 72°C, and an acquisition step of 20 s at 80°C to avoid dimer formation. For fungal densities, real-time PCR conditions consisted in an initial denaturation step of 10 min at 95°C, and 35 cycles comprising a denaturation step of 15 s at 95°C, a primer annealing step of 30 s at 50°C, and a final extension step of 60 s at 72°C. A melt curve was generated for each reaction with a stepwise increase of 0.5°C/s from 60°C to 95°C to check the specificity of PCR amplification. A total of 2,030 soil samples out of 2,173 tested samples were successfully characterized. Obtained values for bacterial and fungal densities were deposited as a Dataverse data set available in recherche.data.gouv.fr (doi.org/10.57745/1Z90HV).

#### Data calibration by post-processing

To enhance the robustness of data comparison, a post-processing treatment was performed by calibration using the master curve method (38). More precisely, three independent replicates of a reference environmental DNA sample and of plasmid DNA standards (10-fold dilution) were added in each experiment. First, the mean of the reference DNA threshold cycles (Ct) of the whole data set was computed. Differences in amplification efficiency between all PCR plates were estimated by computing derivation between the mean of the complete data set reference Ct and the mean reference Ct of each PCR plate. Second, for each plate, the derivation was deducted from the Ct to obtain a corrected Ct. Third, the slope and intercept of a master calibration curve were calculated by using the values (corrected Ct and concentration) of all standards from all experiments. Finally, the number of rDNA copies of each environmental sample was defined based on the corresponding corrected Ct and the master calibration curve parameters.

### Statistical analyses

To characterize spatial variation, a previously described geostatistical method was used to map bacterial and fungal densities and F:B ratios (39) (Tables S2 and S3). Geostatistical modeling was used to study spatial variation. We followed a classical approach as discussed in reference 40. First, a variogram model was fitted to the experimental variogram computed using F:B ratios, and bacterial and fungal densities observed at the sampling sites. Then, we predicted the unsampled positions by the Kriging method using a local neighborhood and “gstat” package (https://cran.r-project.org/web/packages/rgdal/index.html). We tried to fit various authorized variogram models and kept the one that minimized the objective function. Then, we used the results of leave-one-out cross-validation to evaluate the performance of the best fitted geostatistical model by computing the standardized squared prediction errors (41). The relative contributions of five types of explanatory sets of environmental parameters (soil characteristics, land management, climatic conditions, spatial descriptors, and interactions) shaping the patterns of bacterial densities, fungal densities, and F:B ratios were estimated by variance partitioning (3) (Table S3 for climatic conditions). Briefly, the explanatory variables were selected based on the variance inflation factors (VIFs) using “vif” function (“usdm” package and VIF ≤5) to reduce the effect of collinearity of the models and to obtain the most parsimonious models (Table S2). A second selection step based on the Bayesian information criterion and adjusted *R* ^2^ was performed to determine the best explanatory variables using the “regsubset” function (“leaps” package). All quantitative (response and explanatory) data were first standardized to have an approximated Gaussian and homoskedastic residual distribution with a logarithmic or boxcox transformation (“forecast” package). We also detected outliers separately for all response variables using Grubbs’ test strategy (“outliers” package). All identified outliers were removed for further statistical analyses. We used redundancy analysis (RDA) to model variation of overall environmental parameters using the “rda” and “ordiR2step” functions (“vegan” package). Biological indices were used as response variables, and environmental parameters were used as predictor variables. To identify the best indicators, we ran a forward selection to build a model maximizing the adjusted *R* ^2^. A forward selection was run using the “ordiR2step” function and 10,000 permutations maximum. We used “anova.cca” function (vegan package) to estimate the variance explained by the best explanatory environmental variables.

To estimate the multiple comparisons across land uses, we tested if the response variables were normally distributed or approximately so, using the “shapiro.test” function. Depending on the result, we applied a boxcox transformation if the Gaussian assumption was not satisfied. To compute the boxcox transformation from forecast package (42), we estimated the lambda value with the “BoxCox.lambda” function and applied the transformation with the “BoxCox” function. Outliers were tracked using the “grubbs.test” function in the outliers package (https://cran.r-project.org/web/packages/outliers/index.html) for response variables. Once the outliers were removed, we used a nonparametric test with “kruskal” function from “agricolae” package (43) and Bonferroni correction.

## Data Availability

All the data sets used in this paper are openly available. More precisely, measured and corrected values of bacterial and fungal densities as well as fungal:bacterial ratios were deposited as a Dataverse data set available on Recherche Data Gouv. Physicochemical data (doi 10.15454/QSXKGA), land use data (doi 10.15454/QSXKGA), and climate typology data can all be accessed from the previous Dataverse. The script used to collect the data and reproduce the analysis is available via Gitlab.

## SUPPLEMENTAL MATERIAL

Text S1 msphere.00365-23-s0001.docx Detailed values for 16S density, 18S density, and F:B ratios according to defined land uses at a coarse level.

Text S2 msphere.00365-23-s0002.docx Detailed values of 16S density, 18S density, and F:B ratios according to land uses (Forests, Grasslands, Crops, Vineyards & Orchards) at a fine level.

Fig. S1 msphere.00365-23-s0003.docx Mapping and theoretical variograms of soil fungal density at the scale of mainland France.

Fig. S2 msphere.00365-23-s0004.docx Mapping and theoretical variograms of soil bacterial density at the scale of mainland France.

Fig. S3 msphere.00365-23-s0005.docx Distribution of bacterial densities for coarse and more precise land uses.

Fig. S4 msphere.00365-23-s0006.docx Distribution of fungal densities for coarse and more precise land uses.

Table S1 msphere.00365-23-s0007.docx Explained variance values (and associated p-values) of 16S density, 18S density and F:B ratios.

Table S2 msphere.00365-23-s0008.docx Explanatory variable values of variance inflation factors (VIF).

Table S3 msphere.00365-23-s0009.docx Climate types used for the variance partitioning analysis.

ASM does not own the copyrights to Supplemental Material that may be linked to, or accessed through, an article. The authors have granted ASM a non-exclusive, world-wide license to publish the Supplemental Material files. Please contact the corresponding author directly for reuse.
