# Supplementary material for: Biogeographical patterns of the soil fungal:bacterial ratio across France
Source: mSphere. 2023 Sep 27;8(5):e00365-23. doi: 10.1128/msphere.00365-23 (PMC10597451; doi:10.1128/msphere.00365-23)
Supplement: Text S1 — Detailed values for 16S density, 18S density, and F:B ratios according to defined land uses at a coarse level. [file msphere.00365-23-s0001.docx]

**C Djemiel et al. – Supporting Information**

**TEXT S1. Detailed values for 16S density, 18S density and F:B ratios according to defined land uses at a coarse level.**

(A) Summarized data of bacterial (16S) and fungal (18S) densities and fungal:bacterial ratios. (B) Fungal:bacterial ratio density plots.

**A. Summary of 16S, 18S and F:B ratios according to defined land uses at a coarse level.**

| **Metric** | **Value** | **Forests** | **Grasslands** | **Crops** | **Vineyards & Orchards** | **Total** |
| --- | --- | --- | --- | --- | --- | --- |
| 16S density (rDNA copy number per g soil) | Mean | 10,152,416,882.45 | 14,148,261,205.40 | 9,261,730,420.55 | 6,185,488,063.83 | 1.0648E+10 |
|  | Max. | 50,563,802,600.00 | 50,193,403,400.00 | 49,996,752,000.00 | 22,737,066,400.00 | 5.0564E+10 |
|  | Min. | 135,860,300.00 | 472,558,000.00 | 162,826,200.00 | 200,026,200.00 | 135,860,300 |
|  | Median | 6,643,530,250.00 | 11,301,433,500.00 | 6,830,513,500.00 | 4,406,207,400.00 | 7,607,869,200 |
|  | Number of samples | 490.00 | 463.00 | 842.00 | 47.00 | 1,842 |
| 18S density (rDNA copy number per g soil) | Mean | 387,254,671.77 | 353,554,141.31 | 251,970,914.05 | 180,435,083.33 | 312,537,002 |
|  | Max. | 1,527,344,400.00 | 1,484,363,400.00 | 1,485,052,600.00 | 1,199,898,000.00 | 1,527,344,400 |
|  | Min. | 3,074,400.00 | 3,906,000.00 | 3,215,500.00 | 4,078,200.00 | 3,074,400 |
|  | Median | 283,993,600.00 | 267,580,800.00 | 177,979,600.00 | 130,689,400.00 | 221,513,400 |
|  | Number of samples | 503.00 | 489.00 | 847.00 | 48.00 | 1,887 |
| F:B ratio | Mean | 4.45 | 2.52 | 3.05 | 3.07 | 3.29 |
|  | Max. | 12.15 | 11.75 | 11.59 | 10.75 | 12.15 |
|  | Min. | 0.36 | 0.25 | 0.24 | 1.08 | 0.24 |
|  | Median | 3.91 | 2.12 | 2.54 | 2.40 | 2.72 |
|  | Number of samples | 521.00 | 509.00 | 849.00 | 50.00 | 1,929 |

**B. Density plots of fungal:bacterial ratios according to defined land uses at a coarse level.**

Note: hashed red lines represent the fungal:bacterial ratio boundaries ‘1’ and ‘5’.
