# Supplementary material for: Biogeographical patterns of the soil fungal:bacterial ratio across France
Source: mSphere. 2023 Sep 27;8(5):e00365-23. doi: 10.1128/msphere.00365-23 (PMC10597451; doi:10.1128/msphere.00365-23)
Supplement: Text S2 — Detailed values of 16S density, 18S density, and F:B ratios according to land uses (Forests, Grasslands, Crops, Vineyards & Orchards) at a fine level. [file msphere.00365-23-s0002.docx]

**C Djemiel et al. – Supporting Information**

**TEXT S2. Detailed values of 16S density, 18S density and F:B ratios according to land uses (Forests, Grasslands, Crops, Vineyards & Orchards) at a fine level.**

(A-D) Summarized data for each density (16S and 18S) and for fungal:bacterial ratios according to the different land uses. € Density plots of fungal:bacterial ratios).

1. **Summary of 16S, 18S and F:B ratios according to Forests land uses.**

| **Metric** | Land use level 1 | **Forests** | | |
| --- | --- | --- | --- | --- |
|  | Land use level 2 | *Deciduous forests* | *Coniferous forests* | *Mixed forests* |
| 16S density (rDNA copy number per g soil) | Mean | 1.05E+10 | 9.57E+09 | 9.13E+09 |
|  | Max. | 5.06E+10 | 4.39E+10 | 4.71E+10 |
|  | Min. | 1.54E+08 | 1.65E+08 | 1.36E+08 |
|  | Median | 6.46E+09 | 7.10E+09 | 6.08E+09 |
|  | n | 2.77E+02 | 8.40E+01 | 1.21E+02 |
| 18S density (rDNA copy number per g soil) | Mean | 3.78E+08 | 4.13E+08 | 3.98E+08 |
|  | Max. | 1.53E+09 | 1.50E+09 | 1.50E+09 |
|  | Min. | 3.58E+06 | 5.80E+06 | 3.07E+06 |
|  | Median | 2.65E+08 | 3.12E+08 | 3.09E+08 |
|  | n | 281.00 | 83.00 | 131.00 |
| 18:16S ratio | Mean | 4.25 | 5.11 | 4.63 |
|  | Max. | 11.08 | 12.15 | 11.89 |
|  | Min. | 0.36 | 1.08 | 0.87 |
|  | Median | 3.78 | 4.28 | 4.34 |
|  | n | 291.00 | 90.00 | 131.00 |

1. **Summary of 16S, 18S and F:B ratios according to Grasslands land uses.**

| **Metric** | Land use level 1 | **Grasslands** | | | |
| --- | --- | --- | --- | --- | --- |
|  | Land use level 2 | *Permanent grasslands (PG)* | *PG 6-10 years* | *PG > 10 years* | *Unproductive PG* |
| 16S density (rDNA copy number per g soil) | Mean | 1.52E+10 | 1.32E+10 | 1.41E+10 | 1.68E+10 |
|  | Max. | 4.10E+10 | 4.05E+10 | 5.02E+10 | 2.86E+10 |
|  | Min. | 1.91E+09 | 1.63E+09 | 4.73E+08 | 3.41E+09 |
|  | Median | 1.23E+10 | 1.22E+10 | 1.10E+10 | 1.80E+10 |
|  | n | 5.40E+01 | 5.70E+01 | 3.42E+02 | 1.00E+01 |
| 18S density (rDNA copy number per g soil) | Mean | 3.74E+08 | 3.62E+08 | 3.44E+08 | 5.13E+08 |
|  | Max. | 1.00E+09 | 1.07E+09 | 1.48E+09 | 1.47E+09 |
|  | Min. | 5.51E+07 | 1.67E+07 | 3.91E+06 | 1.41E+07 |
|  | Median | 2.88E+08 | 3.29E+08 | 2.56E+08 | 3.49E+08 |
|  | n | 54.00 | 59.00 | 364.00 | 12.00 |
| 18:16S ratio | Mean | 2.85 | 2.72 | 2.46 | 2.09 |
|  | Max. | 11.75 | 8.75 | 11.42 | 5.78 |
|  | Min. | 0.70 | 0.45 | 0.25 | 0.41 |
|  | Median | 2.27 | 2.16 | 2.11 | 1.63 |
|  | n | 54.00 | 61.00 | 381.00 | 13.00 |

1. **Summary of 16S, 18S and F:B ratio values according to Crops land uses**

| **Metric** | Land use level 1 | **Crops** | | |
| --- | --- | --- | --- | --- |
|  | Land use level 2 | *Crops* | *Crops with grassland rotation* | *Fixed fallows* |
| 16S density (rDNA copy number per g soil) | Mean | 8.89E+09 | 1.06E+10 | 8.87E+09 |
|  | Max. | 5.00E+10 | 4.85E+10 | 4.16E+10 |
|  | Min. | 1.63E+08 | 1.79E+08 | 1.65E+08 |
|  | Median | 6.67E+09 | 7.44E+09 | 6.09E+09 |
|  | n | 5.93E+02 | 1.81E+02 | 5.70E+01 |
| 18S density (rDNA copy number per g soil) | Mean | 2.34E+08 | 3.26E+08 | 2.02E+08 |
|  | Max. | 1.49E+09 | 1.45E+09 | 7.25E+08 |
|  | Min. | 3.35E+06 | 1.11E+07 | 3.22E+06 |
|  | Median | 1.73E+08 | 2.36E+08 | 1.39E+08 |
|  | n | 598.00 | 182.00 | 57.00 |
| 18:16S ratio | Mean | 2.96 | 3.30 | 3.05 |
|  | Max. | 11.59 | 9.64 | 11.08 |
|  | Min. | 0.24 | 0.60 | 0.74 |
|  | Median | 2.46 | 2.81 | 2.47 |
|  | n | 600.00 | 182.00 | 56.00 |

1. **Summary of 16S, 18S and F:B ratios according to Vineyards & Orchards land uses.**

| **Metric** | Land use level 1 | **Vineyards & Orchards** | |
| --- | --- | --- | --- |
|  | Land use level 2 | *Vineyards* | *Orchards* |
| 16S density (rDNA copy number per g soil) | Mean | 5.73E+09 | 8.58E+09 |
|  | Max. | 2.27E+10 | 1.72E+10 |
|  | Min. | 2.00E+08 | 1.79E+09 |
|  | Median | 4.02E+09 | 7.37E+09 |
|  | n | 3.60E+01 | 9.00E+00 |
| 18S density (rDNA copy number per g soil) | Mean | 1.63E+08 | 2.70E+08 |
|  | Max. | 1.20E+09 | 7.14E+08 |
|  | Min. | 4.08E+06 | 4.77E+07 |
|  | Median | 1.12E+08 | 1.76E+08 |
|  | n | 37.00 | 9.00 |
| 18:16S ratio | Mean | 3.10 | 3.09 |
|  | Max. | 10.75 | 5.72 |
|  | Min. | 1.08 | 2.06 |
|  | Median | 2.27 | 2.49 |
|  | n | 39.00 | 9.00 |

1. **Density plots of Fungal:Bacterial ratios according to defined land uses.**

Note: hashed red lines represent the Fungal:Bacterial ratio boundaries of 1 and 5.
