# Supplementary material for: Biogeographical patterns of the soil fungal:bacterial ratio across France
Source: mSphere. 2023 Sep 27;8(5):e00365-23. doi: 10.1128/msphere.00365-23 (PMC10597451; doi:10.1128/msphere.00365-23)
Supplement: Fig. S2 — Mapping and theoretical variograms of soil bacterial density at the scale of mainland France. [file msphere.00365-23-s0004.docx]

**FIG S2. ﻿Mapping (A) and theoretical variograms (B) of soil bacterial density at the scale of mainland France.** The colors indicate the extrapolated values expressed as bacterial density (16S rDNA copy number per gram of soil) per soil sample (A). The quality parameters of the model are also detailed. For the variogram (B), points represent the experimental variogram values, and continuous lines the Matern models fitted by the maximum likelihood method.

**
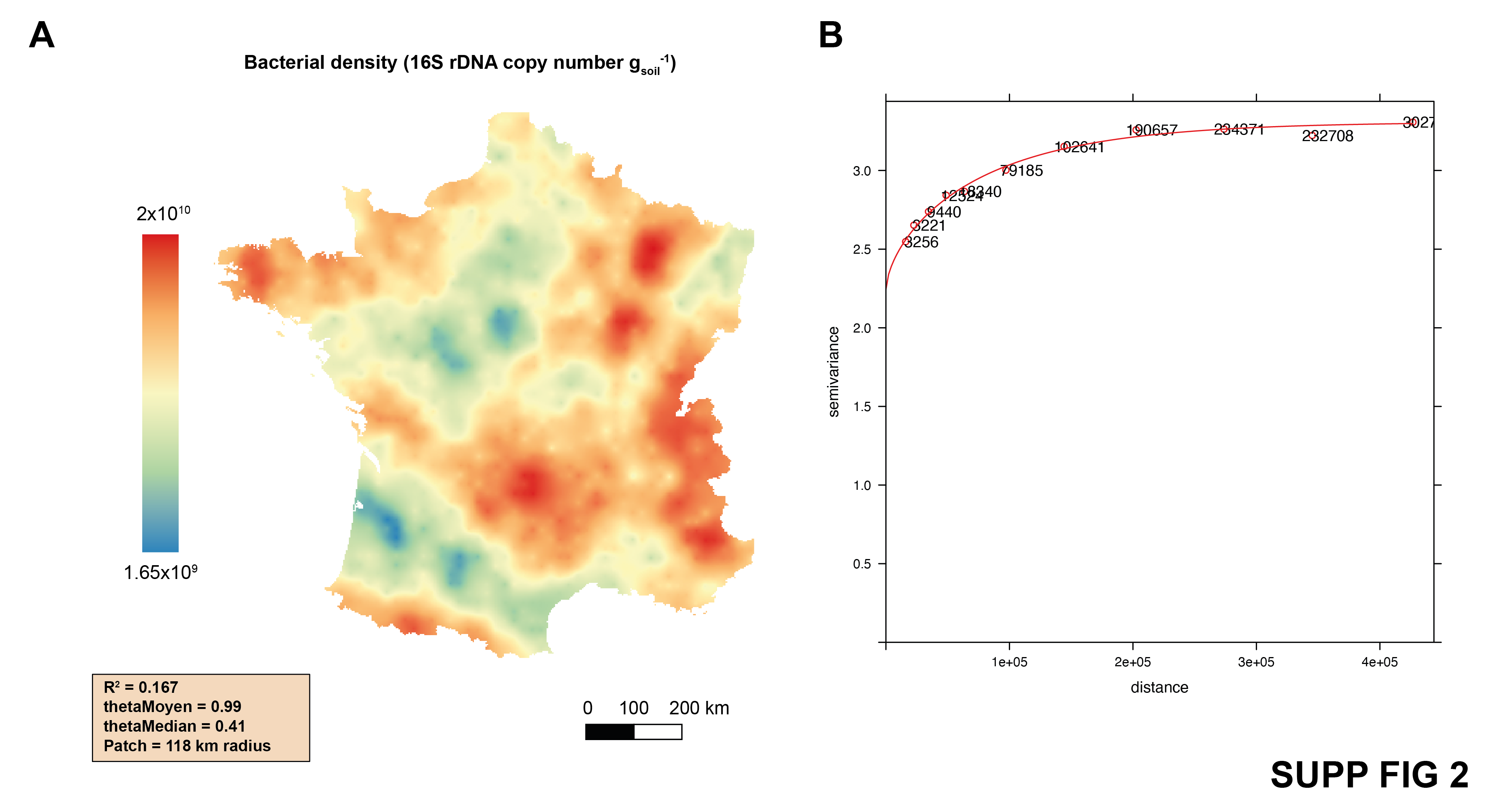
**
