# Supplementary material for: Biogeographical patterns of the soil fungal:bacterial ratio across France
Source: mSphere. 2023 Sep 27;8(5):e00365-23. doi: 10.1128/msphere.00365-23 (PMC10597451; doi:10.1128/msphere.00365-23)
Supplement: Fig. S3 — Distribution of bacterial densities for coarse and more precise land uses. [file msphere.00365-23-s0005.docx]

**FIG S3. ﻿Distribution of bacterial densities for coarse (A) and more precise (B) land uses.** The colors indicate the global land uses (forests, grasslands, crops, vineyards & orchards). Significant differences are indicated after Kruskal-Wallis tests with Bonferroni correction (*p-*value < 0.05).

**
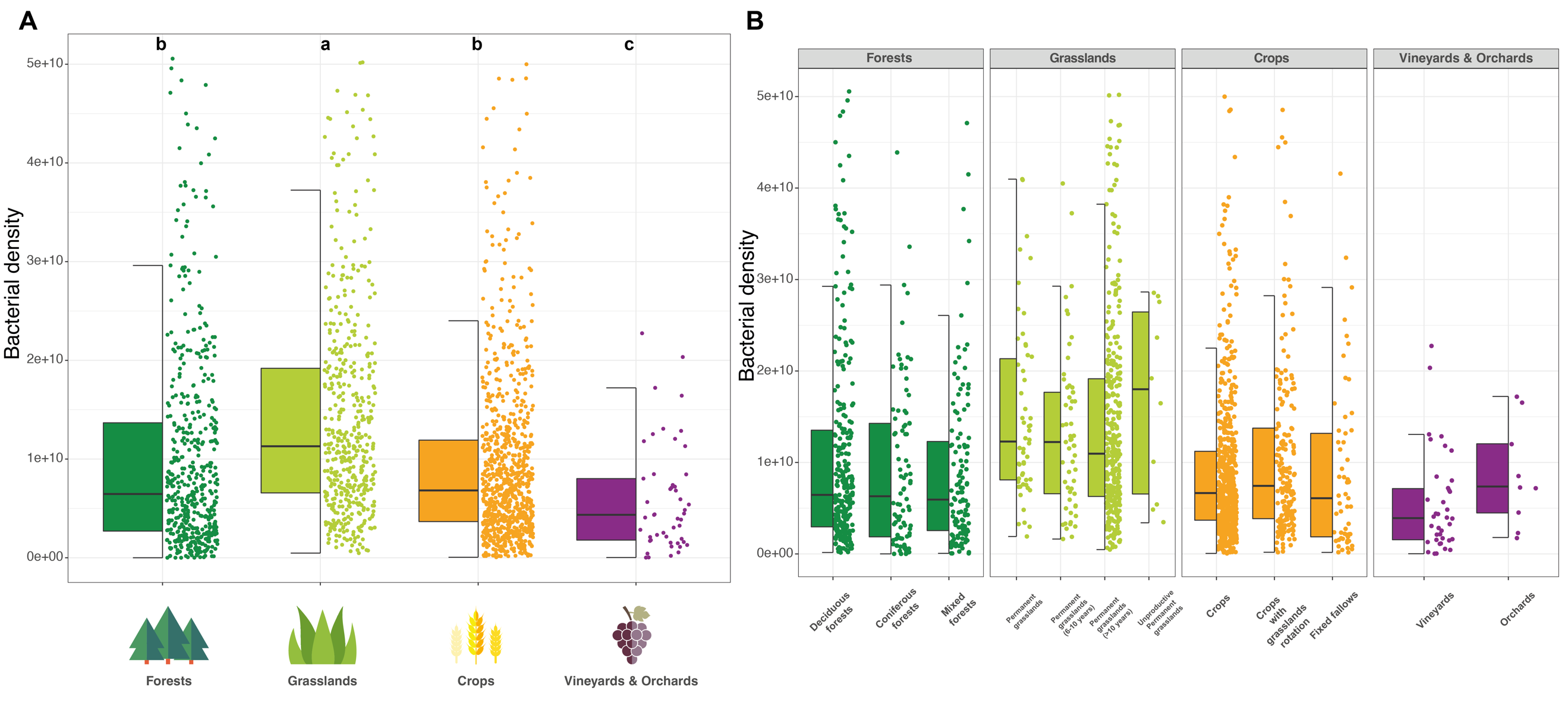
**
