# Supplementary material for: Biogeographical patterns of the soil fungal:bacterial ratio across France
Source: mSphere. 2023 Sep 27;8(5):e00365-23. doi: 10.1128/msphere.00365-23 (PMC10597451; doi:10.1128/msphere.00365-23)
Supplement: Table S1 — Explained variance values (and associated p-values) of 16S density, 18S density and F:B ratios. [file msphere.00365-23-s0007.docx]

|  | **Soil and environmental variables** | **18S:16S ratio** | **16S density** | **18S density** |
| --- | --- | --- | --- | --- |
| Climatic conditions | Climate type | 0.94 (p-value=0.003) | 1.15 (p-value=0.001) | 0.99 (p-value=0.001) |
|  | Median of mean annual precipitation | na | 0.15 (p-value=0.035) | na |
| Land management | Land use | 3.35 (p-value=0.001) | 0.48 (p-value=0.006) | 1.42 (p-value=0.001) |
| Spatial descriptors | Elevation* | 0.44 (p-value=0.003) | na | 0.95 (p-value=0.001) |
|  | Latitude | 0.08 (p-value=0.126) | na | na |
|  | Longitude | 0.38 (p-value=0.003) | na | 0.12 (p-value=0.067) |
| Soil characteristics | Silt | 0.29 (p-value=0.01) | na | 0.53 (p-value=0.001) |
|  | Coarse element content | 0.93 (p-value=0.001) | na | 0.05 (p-value=0.24) |
|  | Bulk dentisty | na | 0.23 (p-value=0.007) | 0.33 (p-value=0.003) |
|  | pH | 4.89 (p-value=0.001) | 1.75 (p-value=0.001) | na |
|  | Organic carbon* | 1.34 (p-value=0.001) | 11.13 (p-value=0.001) | 7.45 (p-value=0.001) |
|  | C:N | 1.03 (p-value=0.001) | 3.25 (p-value=0.001) | 2.24 (p-value=0.001) |
|  | Available phophorus* | 0.27 (p-value=0.006) |  | 0.24 (p-value=0.016) |
|  | Total iron | na | 0.39 (p-value=0.001) | 0.38 (p-value=0.001) |
|  | Total nickel | 0.16 (p-value=0.039) | na | na |
|  | Total copper* | 0.17 (p-value=0.035) | na | na |
|  | Interactions | 14.95 | 22.87 | 20.92 |
|  | Residual | 70.77 | 58.59 | 64.38 |
|  |  |  |  |  |
| *log transformation |  |  |  |  |

**TABLE S1. Explained variance values (and associated *p-*values) of 16S density, 18S density and F:B ratios.**
