# Supplementary material for: Biogeographical patterns of the soil fungal:bacterial ratio across France
Source: mSphere. 2023 Sep 27;8(5):e00365-23. doi: 10.1128/msphere.00365-23 (PMC10597451; doi:10.1128/msphere.00365-23)
Supplement: Table S2 — Explanatory variable values of variance inflation factors (VIF). [file msphere.00365-23-s0008.docx]

| **Variables** | **VIF** |
| --- | --- |
| Median of mean annual potential evapotranspiration | 4.6 |
| Organic carbon* | 3.79 |
| Median of mean annual temperature | 3.78 |
| Latitude | 3.6 |
| Elevation | 3.13 |
| pH | 3.08 |
| Total cadmium* | 2.87 |
| Clay | 2.6 |
| C:N | 2.6 |
| Longitude | 2.5 |
| Total iron | 2.4 |
| Bulk density | 2.21 |
| Total copper* | 2.21 |
| Coarse element content | 2.16 |
| Median of mean annual precipitation | 2.11 |
| Available phosphorus | 1.87 |
| Total lead* | 1.52 |
| Silt | 1.38 |
| Total nickel | 1.33 |
|  |  |
| *log transformation |  |

**TABLE S2. Explanatory variable values of variance inflation factors (VIF).**
