# Supplementary material for: Biogeographical patterns of the soil fungal:bacterial ratio across France
Source: mSphere. 2023 Sep 27;8(5):e00365-23. doi: 10.1128/msphere.00365-23 (PMC10597451; doi:10.1128/msphere.00365-23)
Supplement: Table S3 — Climate types used for the variance partitioning analysis. [file msphere.00365-23-s0009.docx]

| **Climate type** | **Description** |
| --- | --- |
| 1 | Mountain climate |
| 2 | Semi-continental climate and climate of mountain edges |
| 3 | Altered oceanic climate in northern and center plains |
| 4 | Altered oceanic climate |
| 5 | Oceanic climate |
| 6 | Altered Mediterranean climate |
| 7 | Climate of the south-western basin |
| 8 | Mediterranean climate |

**TABLE S3. Climate types used for the variance partitioning analysis.**
